# Predictors of patients’ choices for breast-conserving therapy or mastectomy: a prospective study

**DOI:** 10.1038/sj.bjc.6601835

**Published:** 2004-05-04

**Authors:** S Molenaar, F Oort, M Sprangers, E Rutgers, E Luiten, J Mulder, H de Haes

**Affiliations:** 1 Academic Medical Center, Department of Medical Psychology (J4-401), PO Box 22 660, 1100 DD Amsterdam, The Netherlands; 2 Netherlands Cancer Institute, Plesmanlaan 121, 1066 XC Amsterdam, The Netherlands; 3 St Anna Hospital, PO Box 90, 5660 AB Geldrop, The Netherlands; 4 Medical Spectrum Twente, PO Box 50 000, 7500 KA Enschede, The Netherlands

**Keywords:** breast cancer, treatment decision making, decision aids

## Abstract

A study was undertaken to describe the treatment preferences and choices of patients with breast cancer, and to identify predictors of undergoing breast-conserving therapy (BCT) or mastectomy (MT). Consecutive patients with stage I/II breast cancer were eligible. Information about predictor variables, including socio-demographics, quality of life, patients’ concerns, decision style, decisional conflict and perceived preference of the surgeon was collected at baseline, before decision making and surgery. Patients received standard information (*n*=88) or a decision aid (*n*=92) as a supplement to support decision making. A total of 180 patients participated in the study. In all, 72% decided to have BCT (*n*=123); 28% chose MT (*n*=49). Multivariate analysis showed that what patients perceived to be their surgeons’ preference and the patients’ concerns regarding breast loss and local tumour recurrence were the strongest predictors of treatment preference. Treatment preferences in itself were highly predictive of the treatment decision. The decision aid did not influence treatment choice. The results of this study demonstrate that patients’ concerns and their perceptions of the treatment preferences of the physicians are important factors in patients’ decision making. Adequate information and communication are essential to base treatment decisions on realistic concerns, and the treatment preferences of patients.

In stage I/II breast cancer, breast-conserving therapy (BCT) is equivalent to mastectomy (MT) in terms of survival (Fisher *et al*, 2002; Veronesi *et al*, 2002). Moreover, studies comparing quality of life (QL) after BCT or MT have consistently found an effect on body image but failed to demonstrate overall differences (Kiebert *et al*, 1991; Goodwin *et al*, 2003). In the absence of survival and major QL differences, the treatment decision can be made according to the patient's preference (Goldhirsch *et al*, 2003). Hence, shared decision making is increasingly being proposed as the preferred model of decision making in breast cancer (Charles *et al*, 2003).

Greater understanding of patients’ treatment preferences may establish better and more effective decision making among patients and physicians. Several, mainly North-American studies identified a range of factors influencing breast cancer patients’ treatment preferences. For example, age and education were associated with increased BCT use (Cyran *et al*, 2001; Morrow *et al*, 2001; Nold *et al*, 2001; Gilligan *et al*, 2002; Staradub *et al*, 2002). In addition, patients’ concerns about recurrence, survival, body image and radiation were found to influence treatment preferences (Ward *et al*, 1989; Kotwall *et al*, 1996; Stafford *et al*, 1998; Mastaglia and Kristjanson, 2001; Nold *et al*, 2001). Other, nonpatient factors influencing treatment decisions, include living-area (e.g. rural *vs* urban), type of hospital, and availability of radiation facilities (Mastaglia and Kristjanson, 2001; Morrow *et al*, 2001; Gilligan *et al*, 2002). Moreover, the preference of the surgeon was a major factor in patients’ decision making (Kotwall *et al*, 1996; Stafford *et al*, 1998; Cyran *et al*, 2001; Mastaglia and Kristjanson, 2001; Nold *et al*, 2001).

While the above studies help to understand patients’ treatment preferences, each has a shortcoming. For example, factors influencing decision making in breast cancer have almost exclusively been identified in North-American, retrospective, surveys (Ward *et al*, 1989; Kotwall *et al*, 1996; Stafford *et al*, 1998; Cyran *et al*, 2001; Mastaglia and Kristjanson, 2001; Morrow *et al*, 2001; Nold *et al*, 2001; Gilligan *et al*, 2002). In these studies, unrecognised factors, patient recall and causality are significant problems. Moreover, in the above studies, patients were eligible for BCT and MT on the basis of clinical selection criteria. However, whether patients were explicitly given the opportunity to make a treatment choice was frequently unclear (Stafford *et al*, 1998; Cyran *et al*, 2001; Mastaglia and Kristjanson 2001; Morrow *et al*, 2001; Nold *et al*, 2001; Gilligan *et al*, 2002). In addition, in the above studies, the type and amount of patient education given to patients was frequently not specified.

To improve our knowledge about treatment decision making in breast cancer, the current study used a prospective design, and patients were truly given a choice between BCT and MT. Moreover, all patients received patient education about both treatment options. For this purpose, a decision aid was developed and given to half of the sample. Decision aids have been found to improve patients’ knowledge, diminish decisional conflict and improve realistic explanations (Molenaar *et al*, 2000; O’Connor *et al*, 2003). However, the effect of decision aids on treatment decisions has not been studied to a great extent.

The following research questions are addressed: (1) What are the treatment preferences and treatment decisions of women with stage I/II breast cancer, having a choice between BCT and MT?; (2) Which factors are predictive of patients’ treatment preferences and decisions?; and (3) Does the use of a decision aid, have an impact on the patients’ treatment decision?

## PATIENTS AND METHODS

### Patients

Consecutive patients with proven stage I/II breast cancer, for whom BCT and MT were acceptable treatment options (judged by surgeon) were eligible. Insufficient understanding of Dutch language was the only exclusion criterion used.

### Decision aid

**Figure 1:**
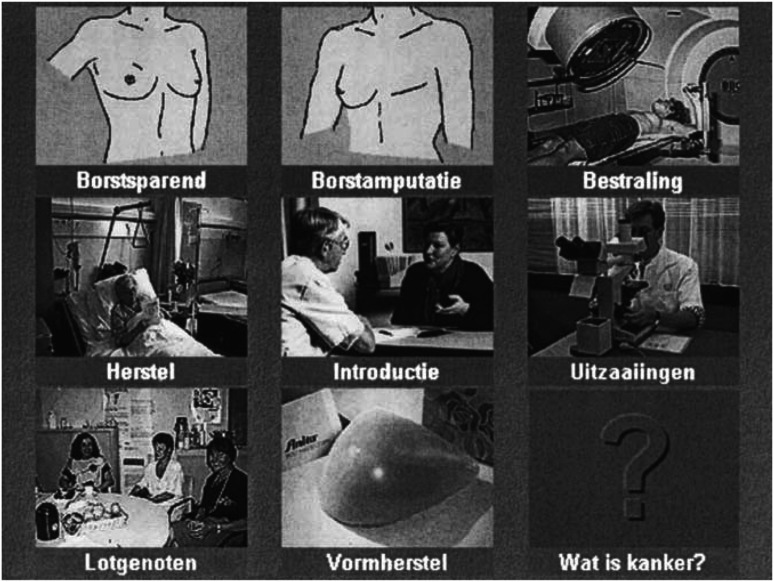
Main menu of decision aid. *Legend*: Borstsparend=breast-conserving therapy; Borstamputatie=mastectomy; Bestraling-radiation therapy; Herstel=recovery; Introductie=introduction; Uitzaaiingen=metastasis; Lotgenoten=fellow patients; Vormherstel=reconstruction; Wat is kanker=what is cancer?

The decision aid was described previously (Molenaar *et al*, 2001a,2001b). Its format is interactive, and its informational content is arranged in a home page which depicts nine modules (Figure 1

). A module is devoted to each treatment option, explaining potential outcomes, benefits and disadvantages.

### Hospitals

Three hospitals participated. One was a cancer hospital (NKI, Amsterdam) and two were general hospitals (St Anna, Geldrop; MST, Enschede). NKI and MST are teaching hospitals with a radiation department on site. Patients from St Anna receive radiation in a neighbouring clinic (5 miles outside Geldrop).

### Study design

The current study stems from a formal evaluation in which the effects of the decision aid on treatment decision, satisfaction and QL were investigated (Molenaar *et al*, 2001b). A standard randomisation design was considered inappropriate as the availability of the decision aid would likely contaminate standard procedures. Therefore, a quasi-experimental design was used. Three inclusion periods of 6 months were created in each hospital (Table 1

**Table 1:** Study design and sample size

**Hospital/Period^a^**	**Period 1**	**Period 2**	**Period 3**	**Total**
1	Standard (*n*=29)	Decision aid (*n*=40)	Standard (*n*=20)	89
2	Decision aid (*n*=16)	Standard (*n*=13)	Decision aid (*n*=17)	46
3	Standard (*n*=14)	Decision aid (*n*=19)	Standard (*n*=12)	45
				
Total	59	72	49	180

a Number of patients per hospital and period given within parentheses.

Total patients per group: Decision aid: *n*=92; Standard information: *n*=88.

). Depending on time period a decision aid was available. Patients in the control periods received standard care (i.e. oral explanations by the surgeon, supplemented with written materials).

### Procedure

The surgeon asked patients to participate once they had discussed diagnosis and treatment. Patients received a written questionnaire, including an informed consent form. This questionnaire was completed before the treatment decision was made (for all patients), and before the decision aid was provided to patients in the decision aid condition. When present, patients used the decision aid within a week at the outpatient department. A nurse was present to provide support, for example with the computer equipment, if necessary. The nurses did not provide patient education. The choice of treatment was made at the day of the patients’ admission to the hospital for surgery for both groups.

### Predictor variables

The following predictor variables were included: socio-demographic patient characteristics; QL; patients’ attitudes towards treatment outcomes; patients’ decision style; decisional conflict; patients’ perceptions of the surgeons’ treatment preferences and having seen the decision aid or not.

*Socio-demographic* characteristics including age, education, having a relationship, children and work (Table 1) were obtained by means of the written questionnaire.

*Generic QL* was assessed with the MOS-20 (Kempen *et al*, 1995), which measures six aspects of QL and an additional energy scale (Table 2

**Table 2:** Patients’ background characteristics at baseline (*n*=180)

**Patient characteristics (*n*=180)**	**Range**	***n* (%)**
Hospital	—	
St. Anna		46 (26)
NKI/AvL		89 (49)
MST		45 (25)
Age (median; min–max)		54; 29–85
Education	—	
<Compulsory		16 (09)
Compulsory		98 (54)
>Compulsory; <university		35 (19)
University		31 (17)
Married/partner; not married/no partner		119 (66); 61 (34)
Lives alone; with spouse/partner		44 (24); 136 (76)
Has child(ren); no child(ren)		148 (82); 32 (18)
Has child(ren) at home; no child(ren) at home		108 (60); 66 (37)^a^
Employed; unemployed		97 (54); 83 (46)
		
Generic QoL (mean; stdev)	0–100	
General health		57.8±23.9
Physical functioning		83.4±22.2
Pain		17.4±20.5
Role functioning		79.7±32.8
Social functioning		84.8±25.1
Psychological functioning		58.7±21.7
Energy		64.8±22.7
		
Breast cancer-specific QoL (mean; stdev)	0–100	
Body image		90.7±14.7
Sexual functioning		24.9±23.2
Breast symptoms		16.7±16.1
Future perspective		47.3±30.4
Concern losing a breast (mean; stdev)	1–10	7.6±3.0
Aversion radiotherapy (mean; stdev)	1–10	6.5±2.5
Concern local recurrence (mean; stdev)	1–5	3.7±1.1
		
Decision style (mean; stdev)	0–100	
Avoidance		21.8±19.0
Deferring responsibility		82.3±17.5
Information seeking		56.2±29.3
Deliberation		88.0±16.0
		
Decisional conflict (mean; stdev)	1–5	
Decision uncertainty		2.8±1.1
Factors contributing to uncertainty		2.5±0.7
		
Patients’ treatment preference	—	
BCT		114 (63)
Unsure		22 (12)
Mastectomy		44 (24)
		
Perceived preference of physician	—	
BCT		85 (47%)
Has no preference		74 (41%)
Mastectomy		19 (11%)

a Missing data for six patients (3%).

) (Stewart and Ware, 1992). QL scores were converted to a 0–100 scale. Higher scores indicate better QL, except for pain where a higher score indicates more pain. The MOS-20 has adequate levels of reliability and validity and was translated into Dutch according to standard forward–backward procedures.

Four subscales of the EORTC QLQ-BR23 were administered to assess *breast cancer-specific QL*. The psychometric quality of this instrument is well established (Sprangers *et al*, 1996). Response scales were transformed to a 0–100 scale. Higher score indicate better body image, sexual functioning, future perspective and having more breast symptoms.

Patients’ attitudes regarding treatment outcomes were assessed. *Patients’ concern about losing a breast* was assessed with a single item (‘To what extent does the prospect of loosing your breast upset you?’) with a 10-point Likert type response format (‘very little’ to ‘very much’). *Aversive feelings towards radiation* were assessed with three items (e.g. ‘To what extent does it bother you that radiation may cause side effects?’) employing a 10-point response scale. The three items were combined to obtain a composite score. *Patients’ concern for local tumour recurrence* was measured with a single question (‘How concerned would you be about the recurrence of a tumour in the breast?’) using a 5-point answering scale (‘very little’ to ‘very much’).

The patients’ *Decision Style* was assessed with The Michigan Assessment of Decision Style (Pierce, 1995). The MADS is developed for decision making in early stage breast cancer, and is well validated. It was translated into Dutch language for the current study by two bilingual individuals, using a forward–backward translation procedure. It covers: (1) avoidance (four items, e.g. ‘I prefer not knowing the possibility that unexpected things could happen to me’); (2) deferring responsibility (three items, e.g. ‘I would follow the recommendations of my physician’); (3) information seeking (four items, e.g. ‘I would spend as much time as I could gathering information’); and (4) deliberation (five items, e.g. ‘I would carefully consider the risks of each option as I was making a choice’). Scores range from 0 to 100, with higher scores indicating more avoidance, deferring, information seeking and deliberation.

Two subscales of the Decisional Conflict Scale were used (O’Connor, 1995). The subscale ‘uncertainty’ refers to the level of uncertainty a patient perceives concerning the decision (three items, e.g. ‘This decision is hard for me to make’). The subscale ‘factors contributing to uncertainty’ (nine items, e.g. ‘I need more advice and information about the treatment alternatives’) measures the extent to which certain factors contribute to decision uncertainty, such as lack of information, unclear values and emotional distress. The DCS has proven to be valid and reliable in different health-care decision-making contexts (O’Connor, 1995). The Dutch version of the DCS was translated, using a forward–backward procedure, and was found to be reliable and valid (Koedoot *et al*, 2001). Decisional conflict ranges from 1 to 5. Higher scores indicate more decisional conflict.

Finally, patients were asked what they thought to be their Surgeons’ treatment preference on a 5-point answering scale (‘definitely BCT’ to ‘definitely MT’).

### Dependent variables

Patients’ baseline Treatment Preference and the actual Treatment Decision were the dependent variables. Treatment preferences were measured at baseline with a single question on a 5-point answering scale (‘certainly BCT’ to ‘certainly MT’). Information about the decision made was collected at 3-months follow-up and checked in the patients’ charts.

### Analysis

Descriptive statistics were used to explore the relationships between patients’ treatment preferences, treatment decisions, and possible predictor variables. Bivariate associations were tested through *χ*^2^ tests, Students’ *t*-tests and analysis of variance.

**Figure 2:**
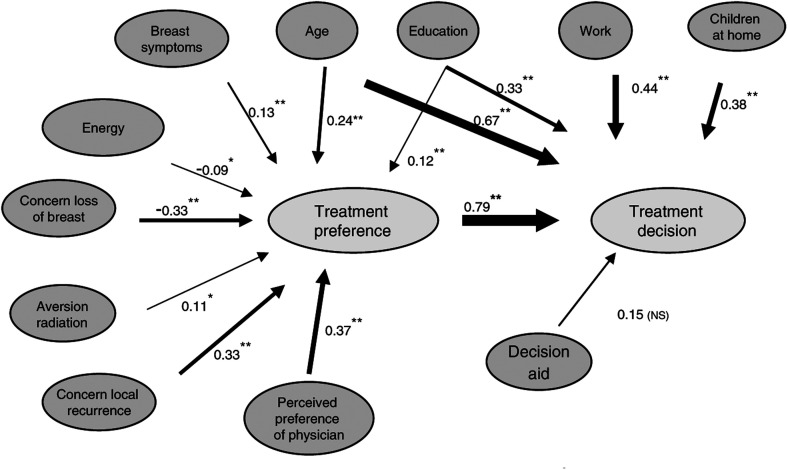
Prediction of treatment preference and decision. Sample size=172; goodness-of-fit: *χ*^2^=8.9, degrees of freedom=8, *P*=0.35; for regression coefficients: ^*^denotes *P*<0.15; ^**^=*P*<0.05. Interpretation: All numbers represent standardised regression coefficients. For example, the −0.33 effect of ’’concern loss of breast’ on ‘treatment preference’ means that an increase in ‘concern’ of one standard deviation (s.d.) is associated with a decrease of 0.33 s.d. in ‘treatment preference’. Moreover, the 0.79 effect of ‘treatment preference’ on ‘treatment decision’ means that an increase of 1 s.d. in ‘treatment preference’ is associated with a 0.79 increase in the probit of ‘treatment decision’. That is, for example, an increase in BCT probability from 50 to 82%. Through ‘treatment preference’, ‘concern loss of breast’ has an indirect effect on ‘treatment decision’ which equals −0.33 × 0.79=−0.26.

The effects of predictor variables, treatment preferences and the decision aid on the treatment decision were further investigated through structural equation modelling (Bollen, 1998). We hypothesised a model in which the predictor variables either have direct effects on the treatment decision, or have indirect effects mediated by treatment preference (Figure 2

). To reduce the number of possible predictor variables, we first carried out linear regression analyses of treatment preference, and logistic regression analyses of treatment decision. The significance level for inclusion of predictors was liberally set at 15%, to not miss any variable that might turn out important in the subsequent structural equation modelling. The fit of the structural equation model to the correlation matrix, consisting of product–moment, biserial and point–biserial correlation coefficients, was evaluated with the *χ*^2^-test of overall goodness-of-fit, that is, the robust weighted least-squares goodness-of-fit test (Satorra, 1992; Muthén and Muthén, 1998). After obtaining a model with good fit, indicated by a nonsignificant *χ*^2^-value, parameter estimates were interpreted. Effects on treatment preference and treatment decision are interpreted in the same way as in ordinary linear regression analysis and probit regression analysis.

## RESULTS

### Patients

In total, 189 women were eligible for the study. Nine decided not to participate (95% response rate). Informed consent was given by all remaining patients (*n*=180). All patients were treated with either BCT or MT between 1996 and 1999. In the decision aid condition, 92 patients were included, and in the standard information condition 88. Eight women were lost to follow-up at 3 months because they were too ill (*n*=3), died (*n*=1) or were no longer motivated (*n*=3). Table 2 presents the patients’ background.

### Treatment preference and treatment decision

BCT was the preferred treatment for 114 patients (63%) (Table 2). MT was preferred by 44 patients (24%). Overall, 22 patients (12%) felt unsure about their preference. Eventually, of 172 women, 123 (72%) decided to have BCT and 49 (28%) MT. Treatment preference and decision were strongly associated (*χ*^2^=80.5; df=4; *P*<0.001; Table 3

**Table 3:** Treatment preference and treatment decision

		**Treatment Decision (*n*=172)**	
		**BCT (*n*; %)**	**MT (*n*; %)**
Treatment preference (n; %)			
Definitely BCT	82 (46)	74 (90)	07 (09)
Probably BCT	32 (18)	26 (81)	03 (09)
Unsure	22 (12)	15 (68)	04 (18)
Probably MT	17 (09)	04 (24)	12 (71)
Definitely MT	27 (15)	04 (15)	23 (85)
			
	180	123 (72%)	49 (28%)

Eight patients were lost at follow-up (see text for details). *χ*^2^=80.5; df=4; *P*<0.001.

). For example, of 82 patients with a preference for BCT at baseline, 90% actually chose this treatment option. Of the patients with a preference for MT, 85% decided to have MT. Of 22 patients who felt unsure about their treatment preference, 15 (68%) decided to undergo BCT and four (18%) MT.

### Predictors of treatment preference

Of socio-demographic variables, age was the only one found to be related to treatment preference (Table 4

**Table 4:** Socio-demographic characteristics, by Treatment Preference and Treatment Decision

	**Treatment preference**				**Treatment decision**		
**Demographic characteristics**	**BCT (*n*=111/114)**	**Unsure (*n*=21/22)**	**MT (*n*=43/44)**	***P***	**BCT (*n*=120/123)**	**MT (47/49)**	***P***
Hospital				0.87			0.50
NKI	66% (59)	12% (11)	21% (19)		71% (60)	29% (25)	
St Anna	63% (29)	11% (05)	26% (12)		78% (35)	22% (10)	
MST	58% (26)	13% (06)	29% (13)		67% (28)	33% (14)	
*Age* (year±s.d.)	52.9 (10.2)	55.8 (10.2)	59.9 (10.6)	<0.01	53.5 (10.0)	58.4 (11.8)	0.07
Education				0.65			0.73
<compulsory	63% (10)	19% (03)	19% (03)		80% (12)	20% (03)	
Compulsory	61% (60)	11% (11)	28% (27)		72% (67)	28% (26)	
Compulsory; <university	69% (24)	17% (06)	14% (05)		73% (24)	27% (09)	
University	65% (20)	07% (02)	29% (09)		65% (20)	36% (11)	
Married/with partner	62% (73)	13% (16)	25% (30)	0.69	74% (83)	27% (30)	0.44
Not married/no partner	67% (41)	10% (06)	23% (14)		68% (40)	32% (19)	
Has child(ren)	62% (91)	12% (18)	26% (39)	0.43	71% (100)	29% (41)	0.72
No child(ren)	72% (23)	13% (04)	16% (05)		74% (23)	26% (08)	
Has child(ren) at home	67% (44)	14% (09)	20% (13)	0.44	69% (44)	31% (20)	0.48
No child(ren) at home	62% (67)	10% (11)	28% (30)		74% (76)	26% (27)	
Lives alone	64% (28)	11% (05)	25% (11)	0.98	67% (29)	33% (14)	0.50
Lives with spouse/partner	63% (86)	13% (17)	24% (33)		73% (94)	27% (35)	
Is employed	68% (66)	09% (09)	23% (22)	0.29	69% (64)	31% (29)	0.40
Not employed	58% (48)	16% (13)	27% (22)		75% (59)	25% (20)	

*P*-values derived from Pearson's *χ*^2^, except for age where *P*-value is derived from *t*-tests and analysis of variances (ANOVA).

); younger patients were more likely to prefer BCT. Most generic and breast cancer-specific QL variables were found to be related to treatment preference (Table 5

**Table 5:** QoL, attitudes, decision style, decisional conflict, perceived physician preference by treatment preference and decision

	**Treatment preference**				**Treatment decision**		
**Variable**	**BCT (*n*=108/114)**	**Unsure (*n*=22)**	**MT (*n*=44)**	***P***	**BCT (*n*=117/123)**	**MT (*n*=46/49)**	***P***
*Generic QoL*							
General health	61.0 (23.6)	53.8 (24.1)	51.8 (23.9)	0.07	59.5 (24.2)	54.1 (23.9)	0.19
Physical functioning	87.5 (18.0)	77.4 (23.6)	95.9 (28.5)	<0.01	85.2 (20.0)	78.9 (27.3)	0.10
Pain	13.5 (17.9)	24.5 (23.0)	24.1 (23.1)	<0.01	15.8 (19.2)	20.0 (22.0)	0.21
Role functioning	84.4 (28.5)	67.0 (42.5)	73.9 (35.7)	0.03	81.1 (31.9)	76.0 (34.6)	0.36
Social functioning	89.3 (20.1)	78.2 (29.5)	76.4 (31.5)	<0.01	86.8 (22.3)	80.0 (31.4)	0.11
Psychological functioning	59.6 (21.6)	59.3 (22.2)	56.1 (22.0)	0.66	58.8 (20.9)	58.8 (24.3)	0.99
Energy	67.8 (23.1)	61.8 (24.2)	58.5 (19.9)	0.06	66.8 (23.2)	61.6 (21.6)	0.18
							
*Breast cancer-specific QoL*							
Body image	92.4 (12.4)	92.0 (14.7)	85.6 (18.8)	0.03	91.2 (14.4)	89.8 (15.9)	0.58
Sexual functioning	28.4 (22.9)	17.4 (18.2)	19.5 (25.0)	0.03	26.8 (22.6)	21.0 (24.5)	0.15
Breast symptoms	13.7 (14.6)	21.6 (22.1)	22.0 (14.8)	<0.01	15.0 (16.0)	20.4 (15.0)	0.05
Future perspective	50.6 (30.3)	39.4 (30.2)	43.2 (30.1)	0.17	47.7 (30.7)	47.6 (28.9)	0.99
							
*Attitudes treatment outcomes*							
Concern loss of breast (1–10)	8.4 (2.5)	6.2 (3.1)	6.3 (3.3)	<0.01	8.0 (2.7)	6.5 (3.3)	<0.01
Aversion radiation (1–10)	6.2 (2.4)	6.6 (2.2)	7.4 (2.6)	0.03	6.4 (2.4)	6.8 (2.6)	0.29
Concern local recurrence (1–5)	3.4 (1.0)	3.8 (0.9)	4.6 (0.6)	<0.01	3.5 (1.0)	4.3 (0.9)	<0.01
							
*Decision style*							
Avoidance	21.0 (18.0)	22.5 (21.6)	23.5 (20.3)	0.75	21.5 (19.4)	21.8 (17.4)	0.94
Defer responsibility	83.0 (17.0)	75.4 (20.0)	84.0 (17.2)	0.14	82.7 (16.7)	81.8 (19.5)	0.77
Information seeking	56.5 (29.1)	57.3 (34.0)	54.8 (28.1)	0.93	56.0 (30.3)	56.3 (26.4)	0.96
Deliberation	87.4 (16.7)	87.3 (17.8)	89.8 (13.2)	0.69	87.4 (16.5)	89.3 (15.1)	0.50
							
*Decisional conflict*							
Decision uncertainty	2.6 (1.1)	3.7 (0.8)	2.7 (1.2)	<0.01	2.8 (1.1)	2.6 (1.1)	0.25
Factors contributing uncertainty	2.5 (0.7)	2.8 (0.8)	2.3 (0.6)	0.06	2.5 (0.8)	2.4 (0.7)	0.54
							
*Perceived preference physician*				<0.01*			<0.01**
Physician prefers BCT	82% (70)	12% (10)	06% (05)		87% (72)	13% (11)	
Physician has no preference	54% (40)	14% (10)	32% (24)		68% (46)	32% (22)	
Physician prefers MT	16% (03)	11% (02)	74% (14)		21% (04)	79% (15)	

* *χ*^2^ 45.9; df 4.

** *χ*^2^ 33.9; df 2.

): patients with a more favourable QL preferred to have BCT. Patients who expressed more concern regarding the loss of a breast also preferred BCT. Conversely, patients who felt more aversion to radiation, and expressed a higher level of concern regarding local tumour recurrence, preferred MT. Wanting to defer responsibility was associated with uncertainty regarding treatment preference. There was no difference in avoidance, information seeking and deliberation between patients favouring BCT or MT. A higher level of decision uncertainty was associated with an uncertain treatment preference. Yet, there was no relation between ‘factors contributing to uncertainty’ and treatment preference. Patients’ treatment preference was related to what patients assumed to be their surgeons’ preferred treatment option. Finally, having had the decision aid was not associated with the treatment preference (Table 6

**Table 6:** Study condition by treatment preference and treatment decision

	**Treatment preference**				**Treatment decision**		
	**BCT (*n*=114)**	**Unsure (*n*=22)**	**MT (*n*=44)**	***P***	**BCT (*n*=123)**	**MT (*n*=49)**	***P***
*Patient education*				0.20			0.37
Decision aid	59% (54)	16% (15)	25% (23)		74% (67)	26% (23)	
Standard information	68% (60)	08% (07)	24% (21)		68% (56)	32% (26)	

*P*-values derived from Pearson's *χ*^2^.

).

### Predictors of the treatment decision

Socio-demographic variables (Table 4) were not associated with the treatment decision in the univariate analysis, nor were QL-variables, except for having breast symptoms (Table 5): patients with more breast symptoms were more likely to want to undergo MT. Concern regarding the loss of a breast was related to BCT. Conversely, concern about local tumour recurrence was associated with MT. Aversion of radiation was not predictive of treatment selection. No relations were found between decision style, decisional conflict and the treatment decision. The treatment decision was related however, to what patients perceived to be their surgeons’ preference. Having seen a decision aid was also not associated with the treatment choice (Table 6).

### A model of treatment preference and decision making

Linear regression analysis predicting treatment preference yielded a first selection (*P*⩽0.15) of eight predictor variables, by both forward and backward selection: age; education; breast symptoms; energy; concern regarding the loss of the breast; concern for local tumour recurrence; aversion to radiation; and perceived preference of the surgeon. Information seeking and deliberation were selected by backward selection only.

Logistic regression analysis predicting the treatment decision yielded a second selection including the hospital involved; age; education; having children; caring for (a) child(ren) at home; being employed; concern about local recurrence; decision uncertainty and treatment preference. We hypothesised that variables of the second selection, that were not in the first selection, have direct effects on treatment decision. Variables that were included in both selections, such as concern about local tumour recurrence, were hypothesised to have indirect effects on treatment decision, mediated by treatment preference.

The overall goodness-of-fit of the resulting model was good (robust weighted least squares; *χ*^2^=12.6; df=11; *P*=0.32). However, inspection of the parameter estimates showed that the regressions of treatment preference on deliberation and information seeking, and the regressions of treatment decision on hospital, decision uncertainty and study-condition were all insignificant (*P*>0.10). Moreover, inspection of residuals and first derivatives showed that in addition to indirect effects, mediated by treatment preference, age and education also had direct effects on treatment decision. Step-by-step modification of the original model led to the model presented in Figure 2. We retained having seen the decision aid or not in the modified model despite its insignificant effect on treatment decision, because of its prime role in the research questions formulated. The fit of this model to the data is good (*χ*^2=^8.9; df=8; *P*=0.35). The model explains 61% of the variance of treatment preference, and 62% of the variance of (the probit of) treatment decision.

What patients thought to be the treatment preference of their surgeon was clearly found to be the best predictor (*β*=0.37) of their own treatment preference. More concern regarding the loss of a breast (*β*=−0.33) was found to be a strong predictor of a preference for BCT. Conversely, more concern for local tumour recurrence (*β*=0.33) and more aversion to radiation (*β*=0.11) were predictive of a preference for MT. Higher age was significantly related to a preference for MT (*β*=0.24). Of QL-related predictors, more breast symptoms (*β*=0.13) and less energy (*β*=−0.09) were predictive of a preference for MT.

An important predictor of the treatment decision was the patients’ previously expressed treatment preference (*β*=0.79). Higher age (*β*=0.67), being employed (*β*=0.44); having to care for children (*β*=0.38) and higher educational level (*β*=0.33), were directly predictive of a choice for MT.

## DISCUSSION

The aim of the present study was to provide insight into the treatment decision-making process of patients with early stage breast cancer. Data were collected prospectively. Patients were clinically eligible for both BCT and MT and were offered to choose between both treatment options. Most patients (63%) preferred to be treated with BCT, whereas one-quarter (24%) preferred MT, and some (12%) felt unsure about their preferred treatment. Subsequently, 90% of the patients chose the type of surgery they initially preferred; 72% chose BCT and 28% MT. This is comparable to the rate of BCT reported by Whelan *et al* (1999). In their study, 73% of patients with a choice opted for BCT after the introduction of a decision aid. Thus, not all women eligible for a choice between BCT and MT want BCT.

Investigating the relative importance of different predictors of treatment preference and the treatment decision resulted in a model with good predictive qualities (Figure 2). What patients thought to be the treatment preference of their physician was found to be the most important predictor of their own treatment preference. Likewise, previous studies also found that physicians are a major factor in the treatment decisions made for breast cancer (Ward *et al*, 1989; Kotwall *et al*, 1996; Stafford *et al*, 1998; Cyran *et al*, 2001; Nold *et al*, 2001). Patients are often inclined to believe that they lack the knowledge and expertise to decide for themselves, that the physician has additional information not (yet) being shared, and that having confidence in the physician means having confidence in their recommendation (Charavel *et al*, 2001; Gurmankin *et al*, 2002). Moreover, patients may believe that being in agreement with the physician is the best guarantee of getting a good treatment. In this respect, there may be a tendency for patients’ to assign their own treatment preference to the physician, as she may want to believe they are in agreement.

Another key finding of our study is that the patients’ concern about local recurrence was found to be an important predictor of a preference and decision for MT. When patients want to minimise their risk for local cancer recurrence, a decision to have MT will indeed address their concern best. However, some patients may inaccurately think that their chance to survive cancer improves when choosing MT. In the long run, this decision may lead to regret when patients learn that the benefit in survival they had wanted, was not gained with MT.

Also, patients’ treatment preferences were strongly influenced by the patients’ concern regarding the loss of a breast, and, to a lesser extent, by their level of breast symptoms, energy and aversion to radiation. These issues related to QL should be addressed foremost in discussions between patients and physicians while deciding upon treatment.

Socio-demographic factors were relatively important in our study. Higher age was predictive of MT. This association has been reported before, even though older women tolerate radiation well, and have excellent control rates (Sakorafas and Tsiotou, 2000). The higher rate of MT observed in older patients may reflect a reluctance of women to undertake the extra visits to the radiation clinic, required to complete BCT, a point of issue also suggested by Staradub *et al* (2002).

Moreover, higher education was associated with MT, while previous studies found a higher level of education to be associated with BCT (Cyran *et al*, 2001; Gilligan *et al*, 2002). It has been suggested that patients with higher education would be better able to understand information regarding recurrence and overall survival and would, as a result, accept BCT more frequently (Cyran *et al*, 2001). The reasons for the association between education and MT in the present study are unknown.

Employed women were more likely to have MT. In a study by Staradub *et al* (2002) an insignificant trend was observed for women with a job to select BCT. However, previous studies found that patients with higher incomes and those having private insurance were more likely to be treated by BCT (Gilligan *et al*, 2002; Morrow *et al*, 2001). Moreover, having to care for children was also associated with MT. Possibly, in the present study, working women and those caring for children were unable to fulfil BCT, which includes 5–6 weeks of daily radiation, due to their duties at work or as a parent. Having to undergo radiation does indeed take a good deal of energy and time from the patient.

The use of a decision aid did not influence the kind of treatment selected. This is a desirable outcome as the aim of the decision aid is to assist patients in the decision-making process, and not to prescribe a course of action. The value of decision aids for breast cancer treatment decision making is increasingly being acknowledged (Goldhirsch *et al*, 2003). Nevertheless, from the current and previous studies it seems clear that patients rely heavily upon their surgeon also for guidance.

Some limitations of our study may be mentioned. Patients’ treatment preferences may be influenced by factors not acknowledged in this study, for example, the size of the tumour; disease stage; patients’ ethnic or cultural background; preferences of a partner or members of the family; fellow patients; and influences of the media, and other health professionals, for example, nurses, radiation physician, family physician.

Moreover, the administration of the baseline questionnaire may have heightened patients’ awareness, and may have influenced certain predictor variables. For example, it may have raised patients’ concern of cancer recurrence or radiation.

It has been frequently documented that many breast cancer patients want to share treatment decision making with their physician (Bruera *et al*, 2002). Given our results, practical suggestions as to how physicians could effectively decide upon treatment with their patients can be made. Although a strong influence of physicians on patients’ decisions does not necessarily mean that patients’ values are disregarded, physicians might try consciously not to impose their own treatment preference on patients. To prevent physicians from exerting too much influence, we suggest that they carefully plan the timing of the communication of their own treatment preference. Prior to giving a treatment preference, surgeons may first have to, neutrally, provide unbiased information concerning both treatments. Decision aids can assist in this information-giving stage. Thereafter, special attention should be given to patients’ concerns and the clarification of their values. Explicitly addressing patients’ understanding of concerns regarding local recurrence and radiation may identify inaccurate beliefs; prevent unfounded fears and ‘irrational’ treatment preferences. It is important to notice that, even when adequately informed, some patients may still favour MT as a means to cope with a more general fear of cancer. Moreover, patients’ concerns for breast loss and breast symptoms are important topics to be addressed in discussions about treatment. After patients’ concerns have sufficiently been discussed, physicians might first only ask patients to express a treatment preference. Rather than providing advice from the start, physicians might then communicate their own treatment preference, or give a treatment recommendation that accounts for the concerns of the individual patient.

Engaging patients in treatment decision making, and refraining from too much influencing the patient is a difficult task (Charavel *et al*, 2001). It does not only require time, but effective communication skills also. The current study helps to select those issues that need attention, and may thus contribute to efficient and effective decision making in early breast cancer.
